# Hybrid and vaccine‐induced immunity against SAR‐CoV‐2 in MS patients on different disease‐modifying therapies

**DOI:** 10.1002/acn3.51727

**Published:** 2023-01-11

Kister I, Curtin R, Pei J, et al. Hybrid and vaccine‐induced immunity against SARS‐CoV‐2 in patients with MS. Ann. Clin. Transl. Neurol. 2022;9(10):1643‐1659. https://onlinelibrary.wiley.com/doi/epdf/10.1002/acn3.51664

In the originally published version of the article, the co‐author's name was erroneously rendered as Igda Stanzin and has now been corrected to Idga Stanzin.

We apologize for this error.

